# Sensorless Modeling of Varying Pulse Width Modulator Resolutions in Three-Phase Induction Motors

**DOI:** 10.1371/journal.pone.0168149

**Published:** 2017-01-11

**Authors:** Matthew David Marko, Glenn Shevach

**Affiliations:** Naval Air Warfare Center Aircraft Division, Joint-Base McGuire-Dix-Lakehurst, Lakehurst NJ 08733, United States of America; Nankai University, CHINA

## Abstract

A sensorless algorithm was developed to predict rotor speeds in an electric three-phase induction motor. This sensorless model requires a measurement of the stator currents and voltages, and the rotor speed is predicted accurately without any mechanical measurement of the rotor speed. A model of an electric vehicle undergoing acceleration was built, and the sensorless prediction of the simulation rotor speed was determined to be robust even in the presence of fluctuating motor parameters and significant sensor errors. Studies were conducted for varying pulse width modulator resolutions, and the sensorless model was accurate for all resolutions of sinusoidal voltage functions.

## Introduction

Electric motors were an important component of industry for well over 100 years, and especially so today. Electric motors are advantageous as they are extremely efficient, environmentally friendly, and are simple to control; much more than mechanical engines that often require complex transmissions. As a result, today electric vehicles (ELV) are becoming more and more available on the road, with hybrids such as the Toyota Prius, plug-in hybrids such as the Chevy Volt, and all-electric cars such as the Tesla becoming much more mainstream on the roads. The US Navy is heavily invested in electric motors for the modern Ford (CVN-78) Class aircraft carrier, with the new Advanced Arresting Gear (AAG) and Electromagnetic Aircraft Launch System (EMALS) utilizing many new motor technologies to improve aircraft launch and recovery. Finally, while subways have used electric motors for well over a century, today advance high-speed trains are being built all over the world. With every passing year, society becomes more and more dependent on the benefits of electric motor technology.

By far the biggest advantage of electric motors is that they are relatively easy to control. Most mechanical engines, whether internal combustion, gas turbine, or steam driven, all require a complex mechanical transmission to give the operator some level of control of the final speed and torque output of the engine. The speed and torque of an electric motor, however, can be controlled by simply adjusting the magnitude and frequency of the power input. One common tool for electric motor control is a Pulse Width Modulator (PWM), which can be used to control the input electric frequency, which can control torque and speed [1]. It is essential to fully understand all of the effects of the PWM in order that a proper control algorithm can be developed for the electric motor.

One important feature of electric motor technology is the ability to have sensorless control. In any motor controller, it is necessary to know the rotor position and speed in order to determine what electric inputs to use for the given application. In an ELV, for example, the cruise control or the driver may want to increase or decrease the motor power depending on the motor speed. An external speed sensor and encoder can be used to measure the rotor speed, but such a system will take up space and weight, as well as be a potential point of failure on an electric motor system. If the encoder is replaced with a sensorless approach that only measures the motor current to determine the speed, the system may be built smaller and more compact. If the sensorless approach is used in combination with a speed encoder, the sensorless system can provide information on the current in real time, which helps to alert the user if there is an issue with the electric motor system. Finally, adding the sensorless approach to complement a traditional encoder adds redundancy to the system in the event of encoder failure.

There have been numerous previous journal publications on sensorless approaches for electric motors [2–18], and even for three-phase motors [19–21]. These modeling approaches, however, have overwhelmingly been analytical, with little focus on the effects of the PWM. The PWM has a series of discrete pulses, with a fixed resolution, where the direct current input can be either on or off. There are several PWM approaches [1], but they result in an approximation of an electric voltage cycle; the greater the PWM resolution, the more the electric current represents a sinusoidal waveform. This effort will seek to model the input current, not as a waveform, but as the discrete components one can expect in a PWM waveform of finite resolution.

## Electric Motor Model

This effort will seek to model a three-phase electric induction motor, created from DC power modified by a PWM, in order to compare and test out any sensorless algorithm. In three-phase electric power, there are three alternating voltage legs offset by 120°, where

$$\begin{array}{ccc} u_{as}\left(t\right) & = & U_{M}\cdot cos(\omega_{f}t),\\ u_{bs}\left(t\right) & = & U_{M}\cdot cos(\omega_{f}t-\frac{2}{3}\pi ),\\ u_{cs}\left(t\right) & = & U_{M}\cdot cos(\omega_{f}t+\frac{2}{3}\pi ), \end{array}$$ (1)

where *U*_*M*_ (volts) is the average voltage amplitude, and *ω*_*f*_ (radians/s) is the electric voltage angular frequency.

In order to simplify the motor analysis, a Park Transform [1] is performed to convert the voltage and current into two separate values instead of three. A Park Transform matrix is defined as

$$\begin{array}{c} {\bar{\bar{P}}}_{T}=(\begin{array}{ccc} \frac{2}{3}cos(\theta ) & \frac{2}{3}cos(\theta -\frac{2}{3}\pi ) & \frac{2}{3}cos(\theta +\frac{2}{3}\pi )\\ \frac{2}{3}sin(\theta ) & \frac{2}{3}sin(\theta -\frac{2}{3}\pi ) & \frac{2}{3}sin(\theta +\frac{2}{3}\pi )\\ \frac{1}{3} & \frac{1}{3} & \frac{1}{3} \end{array}), \end{array}$$

where ${\bar{P}}_{T}$ is the Park Transform matrix, and *θ* is the reference angle. For a stationary reference frame *θ* = 0, which will be used throughout this effort, the Park Transform is simply

$$\begin{array}{c} {\bar{\bar{P}}}_{T}=(\begin{array}{ccc} \frac{2}{3} & -\frac{1}{3} & -\frac{1}{3}\\ 0 & -\frac{1}{\sqrt{3}} & \frac{1}{\sqrt{3}}\\ \frac{1}{3} & \frac{1}{3} & \frac{1}{3} \end{array}), \end{array}$$

and thus this matrix can be applied towards the three-phase voltage vector with the values defined in Eq (1)

$$\begin{array}{c} U_{qdos}^{s}={\bar{\bar{P}}}_{T}\cdot U_{abcs^{\prime }}, \end{array}$$ (2)

where

$$\begin{array}{c} U_{abcs^{\prime }}^{s}=U_{M}\cdot (\begin{array}{c} cos(\omega_{f}t)\\ cos(\omega_{f}t-\frac{2}{3}\pi )\\ cos(\omega_{f}t+\frac{2}{3}\pi ) \end{array}), \end{array}$$

and where

$$\begin{array}{c} U_{qdos^{\prime }}^{s}=(\begin{array}{c} u_{qs}\\ u_{ds}\\ 0 \end{array}). \end{array}$$

Just so long as the three-phase input is balanced, where all three legs are of equal magnitude and separated by 120°, the Park Transform can convert three separate voltage values to two, which simplifies the motor analysis.

The Park Transform is used to convert the three-phase currents into two separate values; referred to as the *quadrature* and *direct* axis components. Throughout the analysis, there are two voltages within the stator *u*_*qs*_ and *u*_*ds*_, two voltages within the rotor *u*_*qr*_ and *u*_*dr*_, two currents within the stator *I*_*qs*_ and *I*_*ds*_, and two currents within the rotor *I*_*qr*_ and *I*_*dr*_. During the motor simulation, all of the voltage and current parameters in the rotor and stator from the previous time-step are known and used in the analysis, along with the true rotor speed. For the sensorless prediction, however, the algorithm can only work with measurable properties such as the voltage and current in the stator; the rotor currents obviously cannot be measured directly, and the goal of the algorithm is to determine the unknown rotor speed.

The next step is to calculate the quadrature and direct axis components of the rotor voltage with the known rotor speed *ω*_*r*_ (rad/s), rotor and stator currents from the previous time-step, as well as the new stator voltages in the next time-step. The zero-axis component is not necessary in this specific model for predicting the torque. The current rates of changes start off as zero, and go into and out of the model

$$\begin{array}{ccc} u_{qr}^{\prime } & = & \left(R_{r}I_{qr}^{\prime }\right)-\omega_{r}\left(L_{lr}^{\prime }I_{dr}^{\prime }+MI_{ds}+MI_{dr}^{\prime }\right)+...\\ & & L_{lr}^{\prime }\frac{dI_{qr}^{\prime }}{dt}+M\frac{dI_{qs}^{\prime }}{dt}+M\frac{dI_{qr}^{\prime }}{dt}, \end{array}$$ (3)

$$\begin{array}{ccc} u_{dr}^{\prime } & = & \left(R_{r}I_{dr}^{\prime }\right)+\omega_{r}\left(L_{lr}^{\prime }I_{qr}^{\prime }+MI_{qs}+MI_{qr}^{\prime }\right)+...\\ & & L_{lr}^{\prime }\frac{dI_{dr}^{\prime }}{dt}+M\frac{dI_{ds}^{\prime }}{dt}+M\frac{dI_{dr}^{\prime }}{dt}, \end{array}$$ (4)

and the rotor voltages can be used to calculate the rate of change of the (quadrature and direct axis) rotor and stator currents

$$\begin{array}{c} \bar{\frac{dI}{dt}}=\bar{\bar{A}}{\bar{I}}_{qd^{\prime }}^{sr}+\bar{B}\cdot \omega_{r}+\bar{\bar{C}}\cdot {\bar{U}}_{qd^{\prime }}^{sr}, \end{array}$$ (5)

where the voltage ${\bar{U}}_{qd^{\prime }}^{sr}$ and current ${\bar{I}}_{qd^{\prime }}^{sr}$ vectors are

$$\begin{array}{c} {\bar{U}}_{qd^{\prime }}^{sr}=(\begin{array}{c} u_{qs}\\ u_{ds}\\ u_{qr}\\ u_{dr} \end{array}), \end{array}$$

$$\begin{array}{c} {\bar{I}}_{qd^{\prime }}^{sr}=(\begin{array}{c} I_{qs}\\ I_{ds}\\ I_{qr}\\ I_{dr} \end{array}), \end{array}$$

and the matrices in Eq (5) are defined as

$$\begin{array}{c} \bar{\bar{A}}=\phi \cdot (\begin{array}{cccc} -L_{RM}\cdot R_{s} & 0 & M\cdot R_{r} & 0\\ 0 & -L_{RM}\cdot R_{s} & 0 & M\cdot R_{r}\\ M\cdot R_{s} & 0 & -L_{SM}\cdot R_{r} & 0\\ 0 & M\cdot R_{s} & 0 & -L_{SM}\cdot R_{r} \end{array}), \end{array}$$

$$\begin{array}{c} \bar{B}=\phi \cdot (\begin{array}{c} -M^{2}\cdot I_{ds}-M\cdot L_{RM}\cdot I_{dr}\\ M^{2}\cdot I_{qs}+M\cdot L_{RM}\cdot I_{qr}\\ L_{SM}\cdot M\cdot I_{ds}+L_{SM}\cdot L_{RM}\cdot I_{dr}\\ -L_{SM}\cdot M\cdot I_{qs}+L_{SM}\cdot L_{RM}\cdot I_{qr} \end{array}), \end{array}$$

$$\begin{array}{c} \bar{\bar{C}}=\phi \cdot (\begin{array}{cccc} L_{RM} & 0 & -M & 0\\ 0 & L_{RM} & 0 & -M\\ -M & 0 & L_{SM} & 0\\ 0 & -M & 0 & L_{SM} \end{array}), \end{array}$$

where the values of these parameters are defined as

$$\begin{array}{c} \phi =\frac{1}{L_{SM}L_{RM}-M^{2}}, \end{array}$$ (6)

$$\begin{array}{c} L_{SM}=L_{ls}+M, \end{array}$$ (7)

$$\begin{array}{c} L_{RM}=L_{lr}+M, \end{array}$$ (8)

$$\begin{array}{c} M=\frac{3}{2}L_{ms}, \end{array}$$ (9)

where *R*_*r*_ (Ω) is the rotor resistance, *R*_*s*_ (Ω) is the stator resistance, *L*_*ms*_ (Henry) is the stator magnetizing inductance, *L*_*ls*_ (Henry) is the stator leakage inductance, and *L*_*lr*_ (Henry) is the rotor leakage inductance.

The change in current is calculated simply by

$$\begin{array}{c} I(t)=I_{0}+\delta t\cdot \frac{dI}{dt}. \end{array}$$ (10)

The model uses iteration to converge on both the previous time-step current *I*_0_ and current rate-of-change $\frac{dI}{dt}$. The initial guess for the new time-step is simply the current data from the previous time-step.

Throughout the motor simulation, the motor parameters are offset by a random fluctuation. In any practical application, the motor parameters will always fluctuate from the published or measured values, and therefore in order to test the robustness of the model, the simulation will offset the simulated motor parameters by a specified amount (typically 5%); these offset parameters will *not* be used within the sensorless algorithm. The motor fluctuation equation is

$$\begin{array}{c} \Theta =\Theta_{0}\cdot \left[1+\frac{\epsilon }{100}\left(2\delta -1\right)\right], \end{array}$$ (11)

where Θ is a transient arbitrary motor property, Θ_0_ is a arbitrary motor property as it is specified, *ϵ* (%) is the percent random error that will be simulated, and *δ* is a random number ranging from 0 to 1.

Finally, once the rotor and stator current are determined, the motor torque *T*_*motor*_ (N ⋅ m) can be calculated

$$\begin{array}{c} T_{motor}=\frac{3P}{4}\cdot M\cdot \left(I_{qs}I_{dr}^{\prime }-I_{ds}I_{qr}^{\prime }\right), \end{array}$$ (12)

where *P* is the number of poles in the motor. This torque can be used to determine the change in speed on the rotor and ultimately the vehicle powered by the motor.

## Sensorless Algorithm

All of the equations in the previous section are to accurately model a three-phase electric motor for a given electrical input; all of the parameters including the motor parameter fluctuation, the rotor speed, and the rotor voltages and currents were used in the motor model. The goal of the sensorless algorithm is, however, to determine the rotor speed that is unknown to the motor controller. It is obviously impossible to measure the rotor voltage and current in real time, but the stator currents can be measured and compared with the controller input voltages. The goal of the sensorless algorithm will be to attempt to determine the unknown rotor speed, rotor voltage, and rotor current, from the measured and known stator input voltage out of the PWM, as well as the stator current (which can be physically measured in real time) determined by the motor model.

The sensorless algorithm first will attempt to determine the rotor currents; this can be determined from the stator flux. The rate of change of the stator flux can be calculated from known stator voltages and currents

$$\begin{array}{ccc} \frac{d\Psi_{qs}}{dt} & = & u_{qs}-r_{s}\cdot I_{qs}, \end{array}$$ (13)

$$\begin{array}{ccc} \frac{d\Psi_{ds}}{dt} & = & u_{ds}-r_{s}\cdot I_{ds}, \end{array}$$ (14)

and the stator flux can be determined from the calculated rate of change

$$\begin{array}{ccc} \Psi_{qs} & = & \Psi_{qs,0}+dt\cdot \frac{d\Psi_{qs}}{dt}, \end{array}$$ (15)

$$\begin{array}{ccc} \Psi_{ds} & = & \Psi_{ds,0}+dt\cdot \frac{d\Psi_{ds}}{dt}, \end{array}$$ (16)

and the flux is tracked throughout the simulation. At any point of no voltage, such as at the start of the simulation, the flux is initially assumed to be zero. Once the stator flux is predicted, the rotor current can be calculated

$$\begin{array}{ccc} {\hat{I}}_{qr} & = & \frac{\Psi_{qs}-\left(M+L_{ls}\right)\cdot I_{qs}}{M}, \end{array}$$ (17)

$$\begin{array}{ccc} {\hat{I}}_{dr} & = & \frac{\Psi_{ds}-\left(M+L_{ls}\right)\cdot I_{ds}}{M}, \end{array}$$ (18)

and the rotor current predicted value is tracked throughout the simulation. Unlike the stator flux, the rotor flux is not useful in this sensorless algorithm, but it can nevertheless be determined with the rotor currents

$$\begin{array}{ccc} {\hat{\Psi }}_{qr} & = & \left(M+L_{ls}\right)\cdot {\hat{I}}_{qr}+M\cdot I_{qs}, \end{array}$$ (19)

$$\begin{array}{ccc} {\hat{\Psi }}_{dr} & = & \left(M+L_{ls}\right)\cdot {\hat{I}}_{dr}+M\cdot I_{ds}. \end{array}$$ (20)

Looking at Eq (5), a function for the rotor speed ${\hat{\omega }}_{r}$ (rad/s) can be found provided one knows all of the voltages. The stator voltages are known, but the rotor voltages need to be predicted. If the rotor speed is known, the rotor voltages can be calculated with Eqs (3) and (4), but since the rotor voltage is not known, the sensorless algorithm splits these equations into terms that are and are not a function of the rotor speed

$$\begin{array}{ccc} {\hat{u}}_{qr}^{\prime } & = & {\hat{u}}_{qr1}^{\prime }+{\hat{u}}_{qr2}^{\prime }\cdot {\hat{\omega }}_{r},\\ {\hat{u}}_{qr1}^{\prime } & = & \left(R_{r}\cdot {\hat{I}}_{qr}^{\prime }\right)+L_{lr}^{\prime }\cdot \frac{d{\hat{I}}_{qr}^{\prime }}{dt}+M\cdot \frac{dI_{qs}^{\prime }}{dt}+M\cdot \frac{d{\hat{I}}_{qr}^{\prime }}{dt},\\ {\hat{u}}_{qr2}^{\prime } & = & -(L_{lr}^{\prime }\cdot {\hat{I}}_{dr}^{\prime }+M\cdot I_{ds}+M\cdot {\hat{I}}_{dr}^{\prime }), \end{array}$$ (21)

and

$$\begin{array}{ccc} {\hat{u}}_{dr}^{\prime } & = & {\hat{u}}_{dr1}^{\prime }+{\hat{u}}_{dr2}^{\prime }\cdot {\hat{\omega }}_{r},\\ {\hat{u}}_{dr1}^{\prime } & = & \left(R_{r}\cdot {\hat{I}}_{dr}^{\prime }\right)+L_{lr}^{\prime }\cdot \frac{d{\hat{I}}_{qr}^{\prime }}{dt}+M\cdot \frac{dI_{qs}^{\prime }}{dt}+M\cdot \frac{d{\hat{I}}_{qr}^{\prime }}{dt},\\ {\hat{u}}_{dr2}^{\prime } & = & -(L_{lr}^{\prime }\cdot {\hat{I}}_{dr}^{\prime }+M\cdot I_{qs}+M\cdot {\hat{I}}_{qr}^{\prime }). \end{array}$$ (22)

These terms can be used to find new voltage vectors

$$\begin{array}{c} {\bar{U}}_{qd1^{\prime }}^{sr}=(\begin{array}{c} u_{qs}\\ u_{ds}\\ {\hat{u}}_{qr1}\\ {\hat{u}}_{dr1} \end{array}), \end{array}$$

and

$$\begin{array}{c} {\bar{U}}_{qd2^{\prime }}^{sr}=(\begin{array}{c} 0\\ 0\\ {\hat{u}}_{qr2}\\ {\hat{u}}_{dr2} \end{array}), \end{array}$$

and these new voltage vectors can be plugged into the equation for the rate of change of the current (Eq (5))

$$\begin{array}{c} \bar{\frac{dI}{dt}}=\bar{\bar{A}}\cdot {\bar{I}}_{qd^{\prime }}^{sr}+\bar{B}\cdot {\hat{\omega }}_{r}+\bar{\bar{C}}\cdot {\bar{U}}_{qd1^{\prime }}^{sr}+\bar{\bar{C}}\cdot {\bar{U}}_{qd2^{\prime }}^{sr}\cdot {\hat{\omega }}_{r}, \end{array}$$ (23)

which can be separated into

$$\begin{array}{c} \bar{\frac{dI}{dt}}-\bar{\bar{A}}\cdot {\bar{I}}_{qd^{\prime }}^{sr}-\bar{\bar{C}}\cdot {\bar{U}}_{qd1^{\prime }}^{sr}=\left(\bar{B}+\bar{\bar{C}}\cdot {\bar{U}}_{qd2^{\prime }}^{sr}\right)\cdot {\hat{\omega }}_{r}, \end{array}$$ (24)

and thus the rotor speed can be separated out as

$$\begin{array}{c} {\hat{\omega }}_{r}=\frac{\bar{\frac{dI}{dt}}-\bar{\bar{A}}\cdot {\bar{I}}_{qd^{\prime }}^{sr}-\bar{\bar{C}}\cdot {\bar{U}}_{qd1^{\prime }}^{sr}}{\bar{B}+\bar{\bar{C}}\cdot {\bar{U}}_{qd2^{\prime }}^{sr}}. \end{array}$$ (25)

## Model Parameters

This effort is not seeking to model a particular ELV; only an arbitrary scenario to demonstrate this sensorless algorithm and how it’s accuracy is immune to both variation in the PWM resolution, as well as random fluctuations of the voltage and current sensors. For the sake of this arbitrary simulation, an ELV will be modeled, where the controller will ramp up the speed from 0 to 100 mph over 5 minutes, in 30 second increments of 10 mph. The speed will be predicted based solely on measurements of the stator voltages and currents, and the overall distance traveled will be compared to the integrated velocity predicted by the stator voltages and currents. The voltage will be set to the maximum 10 kV when accelerating, and a cruising voltage of 2.5 kV will be applied when the predicted voltage is within 5% of the cruising speed.

In addition, a random hill function is applied to adjust the gravity force accelerating or decelerating the EVL; the standard deviation of the random hill grade is ±17.3184°, while the maximum hill angle possible is ±30°. When the angle is known, a vehicle (and thus rotor) acceleration / deceleration is applied from the force of gravity

$$\begin{array}{ccc} a_{hill} & = & g\cdot sin(\theta ), \end{array}$$ (26)

where *θ* is the random hill angle, *g* is the gravitational acceleration of 9.81 m/s^2^, and *a*_*hill*_ (m/s^2^) is the accelation / deceleration of the ELV due to the random hills on the road. This can be modified to represent an angular acceleration

$$\begin{array}{ccc} {\dot{\omega }}_{hill} & = & \frac{2\cdot M_{car}\cdot g}{D_{W}}\cdot sin(\theta ), \end{array}$$ (27)

where *M*_*car*_ (kg) is the mass of the ELV, *D*_*W*_ (m) is the diameter of the tire, and ${\dot{\omega }}_{hill}$ (rad/s^2^) is the angular acceleration of the wheel axial due to the randomly fluctuating hill.

Finally, the model will use a drag coefficient, applying a decelerating force proportional to the square of the forward speed

$$\begin{array}{ccc} {\dot{\omega }}_{drag} & = & K_{drag}\cdot 2\cdot Dw\cdot {\left(\frac{\omega_{RPM}\cdot \pi }{60}\right)}^{2}, \end{array}$$ (28)

where *ω*_*RPM*_ is the rotor speed in revolutions per minute, and *K*_*drag*_ is an arbitary coefficient for drag

$$\begin{array}{ccc} K_{drag} & = & C_{D}\cdot \rho \cdot A\frac{1}{M_{car}}, \end{array}$$ (29)

where *ρ* (kg/m^3^) is the density, *A* (m^2^) is the surface area, and *C*_*D*_ is the dimensionless drag coefficient; for the vehicle’s shape, *C*_*D*_ is a function of the dimensionless Reynolds Number. In this simulation, the drag coefficient is set at *K*_*drag*_ = 2 N/RPM^2^.

**Fig 1:**
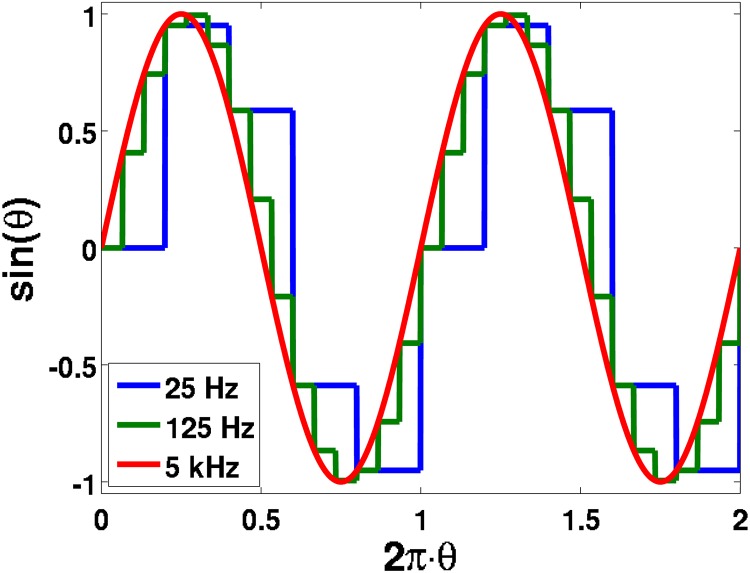
The sinosoidal function simulated for the output of a 25 Hz, 75 Hz, and 5 kHz PWM function, all with a 200 *μ*s time-step.

A parametric study of the sensorless model under varying conditions was performed. Each 5 minute run was conducted with a consistent time step of 5 milliseconds, but with varying PWM resolutions that ran from 20 Hz to 200 Hz in increments of 20 Hz; varying PWM angular functions are simulated in Fig 1. These PWM resolutions and the time-step were selected due to the simulated electric motor frequency of 6 Hz. To further test the robustness of the sensorless algorithm, after every minute of simulation, the overall motor parameters would fluctuate randomly by 1%; the adjusted motor parameters would be unknown to the sensorless prediction algorithm. Finally, the error range was varied in 5% to 45% in increments of 10%; this percent error was randomly applied to the measured stator voltages and currents at each time step, to validate the robustness of the prediction method. These fifty independent computation tests were validated by comparing the integrated simulated and predicted distances traveled at every time step increment.

## Model Results

**Fig 2:**
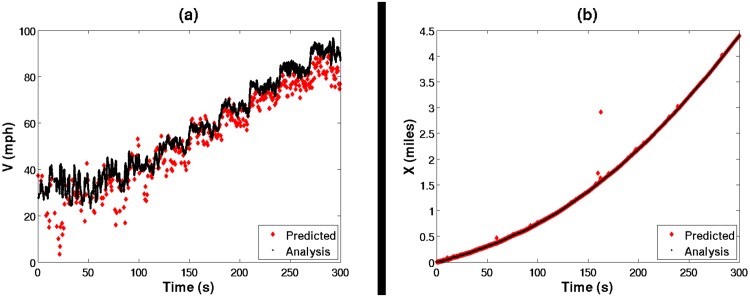
*Predicted* with the sensorless algorithm, and simulated motor *analysis* results of both (a) the EVL velocity, as well as (b) the integrated distance traveled.

In each of these fifty simulations, at each of the 60,000 time steps, the distance traveled was calculated by numerically integrating the true analytical velocity. A second, predicted distance was determined by numerically integrating the velocity predicted by the measured stator voltage and current. It is qualitatively obvious in Fig 2a that the predicted velocity at each specific time-step will vary considerably to the actual velocity; however, the numerically integrated distance traveled was observed in Fig 2b to match remarkably.

At every time-step, the percent error of the distance traveled was calculated. The sensorless algorithm is considered to have matched if the predicted distance traveled varied from the actual distance traveled by more than 0.01%. As observed in Table 1, the predicted integrated distance traveled matches remarkably; only a few percentage points of time-steps varied more than this threshhold! There was an expected increase in error with increasing voltage and current sensor fluctuation; this error was very minor, as the prediction method managed to correct itself. In addition, the sensorless prediction method had no discernable change in error rate with differing PWM fluctuations, even as the PWM resolution was increased by an order of magnitude. This parametric study has demonstrated the robustness of this prediction method for determining the velocity and distance traveled by a vehicle, without having direct measurements of the wheel speed.

**Table 1:** Percentage (%) of data points where the prediction method error was greater than 0.01%, for varying PWM frequency (Hz) and percent voltage and current sensor error.

	5%	15%	25%	35%	45%
**20 Hz**	1.3583	2.6017	3.4817	6.3183	6.4067
**40 Hz**	1.15	2.57	3.6283	4.2783	6.005
**60 Hz**	1.8167	2.3033	4.575	6.4133	6.6767
**80 Hz**	0.96	2.655	3.5183	5.2133	6.49
**100 Hz**	1.6133	2.705	4.5483	4.9533	8.1517
**120 Hz**	1.9217	3.09	3.7267	5.0533	6.6983
**140 Hz**	0.92	3.2983	3.985	5.6767	7.4033
**160 Hz**	1.5217	3.1133	3.7183	4.8867	6.785
**180 Hz**	1.2283	3.7833	3.78	5.2717	7.3
**200 Hz**	0.885	3.18	4.205	5.5	6.8183

## Conclusion

This effort succeeded in first building a numerical motor model [1] to replicate the controls on an arbitrary ELV traveling through random hilly conditions. A sensorless rotor-speed prediction algorithm was then developed, which used analytical equations [1, 2, 4, 19–21] to predict the change in stator flux, which could be used to predict (based on the previous time step flux) the stator flux. This flux can be used to predict the rotor currents, which are ultimately used to predict the rotor voltages, flux, and most importantly the unknown rotor speed. This method allows the motor controller to have redundancy on the rotor speed sensor, or even disregard it if the size and weight are an issue; the only measurements needed were the stator voltage and currents.

Of course, sensorless motor modeling is nothing new [1–21]. What makes this technique unique is the emphasis on AC signals that are not quite sinusoidal due to limits in the resolution of the PWM. This sensorless prediction method has been validated to be extremely robust even in the presence of unknown motor fluctuations, with voltage and current sensors fluctuating up to 45% of the true value, and it can be used for any PWM output. Provided the sensorless time-step is small enough, the sensorless model can work with any alternating electrical current function provided it is separated into three separate legs. As a result, this technique, which only requires a computer and a voltage and current sensor in the motor’s stator, can contribute greatly to ensuring greater reliability and safety for critical applications of three-phase induction motor controllers.

## Supporting Information

S1 File MatLab Source Code. A PDF file of all of the MatLab source code used in this study.
(PDF)
