# Supplementary material for: Sensorless Modeling of Varying Pulse Width Modulator Resolutions in Three-Phase Induction Motors
Source: PLoS One. 2017 Jan 11;12(1):e0168149. doi: 10.1371/journal.pone.0168149 (PMC5226809; doi:10.1371/journal.pone.0168149)
Supplement: S1 File — A PDF file of all of the MatLab source code used in this study. (PDF) [file pone.0168149.s001.pdf]

parametric.m

```
clear

RawFct(10,1,5,5,1.1,6,'PWM10Error5');
run
RawFct(20,1,5,5,1.1,6,'PWM20Error5');
run
RawFct(40,1,5,5,1.1,6,'PWM40Error5');
run
RawFct(60,1,5,5,1.1,6,'PWM60Error5');
run
RawFct(80,1,5,5,1.1,6,'PWM80Error5');
run
RawFct(100,1,5,5,1.1,6,'PWM100Error5');
run
RawFct(120,1,5,5,1.1,6,'PWM120Error5');
run
RawFct(140,1,5,5,1.1,6,'PWM140Error5');
run
RawFct(160,1,5,5,1.1,6,'PWM160Error5');
run
RawFct(180,1,5,5,1.1,6,'PWM180Error5');
run
RawFct(200,1,5,5,1.1,6,'PWM200Error5');
run

RawFct(10,1,15,15,1.1,6,'PWM10Error15');
run
RawFct(20,1,15,15,1.1,6,'PWM20Error15');
run
RawFct(40,1,15,15,1.1,6,'PWM40Error15');
run
RawFct(60,1,15,15,1.1,6,'PWM60Error15');
run
RawFct(80,1,15,15,1.1,6,'PWM80Error15');
run
RawFct(100,1,15,15,1.1,6,'PWM100Error15');
run
```

```
RawFct(120,1,15,15,1.1,6,'PWM120Error15');
run
RawFct(140,1,15,15,1.1,6,'PWM140Error15');
run
RawFct(160,1,15,15,1.1,6,'PWM160Error15');
run
RawFct(180,1,15,15,1.1,6,'PWM180Error15');
run
RawFct(200,1,15,15,1.1,6,'PWM200Error15');
run

RawFct(10,1,25,25,1.1,6,'PWM10Error25');
run
RawFct(20,1,25,25,1.1,6,'PWM20Error25');
run
RawFct(40,1,25,25,1.1,6,'PWM40Error25');
run
RawFct(60,1,25,25,1.1,6,'PWM60Error25');
run
RawFct(80,1,25,25,1.1,6,'PWM80Error25');
run
RawFct(100,1,25,25,1.1,6,'PWM100Error25');
run
RawFct(120,1,25,25,1.1,6,'PWM120Error25');
run
RawFct(140,1,25,25,1.1,6,'PWM140Error25');
run
RawFct(160,1,25,25,1.1,6,'PWM160Error25');
run
RawFct(180,1,25,25,1.1,6,'PWM180Error25');
run
RawFct(200,1,25,25,1.1,6,'PWM200Error25');
run

RawFct(10,1,35,35,1.1,6,'PWM10Error35');
run
RawFct(20,1,35,35,1.1,6,'PWM20Error35');
run
RawFct(40,1,35,35,1.1,6,'PWM40Error35');
run
```

```
RawFct(60,1,35,35,1.1,6,'PWM60Error35');
run
RawFct(80,1,35,35,1.1,6,'PWM80Error35');
run
RawFct(100,1,35,35,1.1,6,'PWM100Error35');
run
RawFct(120,1,35,35,1.1,6,'PWM120Error35');
run
RawFct(140,1,35,35,1.1,6,'PWM140Error35');
run
RawFct(160,1,35,35,1.1,6,'PWM160Error35');
run
RawFct(180,1,35,35,1.1,6,'PWM180Error35');
run
RawFct(200,1,35,35,1.1,6,'PWM200Error35');
run

RawFct(10,1,45,45,1.1,6,'PWM10Error45');
run
RawFct(20,1,45,45,1.1,6,'PWM20Error45');
run
RawFct(40,1,45,45,1.1,6,'PWM40Error45');
run
RawFct(60,1,45,45,1.1,6,'PWM60Error45');
run
RawFct(80,1,45,45,1.1,6,'PWM80Error45');
run
RawFct(100,1,45,45,1.1,6,'PWM100Error45');
run
RawFct(120,1,45,45,1.1,6,'PWM120Error45');
run
RawFct(140,1,45,45,1.1,6,'PWM140Error45');
run
RawFct(160,1,45,45,1.1,6,'PWM160Error45');
run
RawFct(180,1,45,45,1.1,6,'PWM180Error45');
run
RawFct(200,1,45,45,1.1,6,'PWM200Error45');
run
```

run.m

```
clear all
close all
clc

tic

% RawFct(200,1,15,15,1.1,10,'Crap');
load RawData

Position=[200 200 900 800];
TitleSz=24; LblSz=20; AxSz=16;
NL=sprintf('\n');

X0=X00; V0=V00; X0ssl=X00; V0ssl=V00;
TimeFct=(0:(Nct-1))*dt;
t=0;

Xfct=zeros(1,Nct); Xfct(1)=X0;
XfctSSL=zeros(1,Nct); XfctSSL(1)=X0ssl;
Vfct=zeros(1,Nct); Vfct(1)=V0;
RPMfct=zeros(1,Nct);
SSLfct=zeros(1,Nct);
ErrorX=zeros(1,Nct); ErrorV=zeros(1,Nct);
angFct=zeros(1,Nct);
HillFct=zeros(1,Nct);
Afct=zeros(1,Nct); A0=0;
Ifct=zeros(Nct,4);
VoltFct=zeros(1,Nct); Volt=0;
TorqueRealFct=zeros(1,Nct);
IabcSfct=zeros(Nct,3); Iabcs0=zeros(3,1);
IpwdFct=zeros(PWM,4);
dIabcsFct=zeros(Nct,3);
UqdFct=zeros(Nct,4); UqdFctPWD=zeros(PWM,4);
UabcSfct=zeros(Nct,3);
fFct=zeros(1,Nct);
slipFct=zeros(1,Nct);
FluxQDfct=zeros(Nct,4); FluxQD=zeros(1,4); FluxQD0=FluxQD; dFluxQD=zer
```

```
Ig=zeros(4,1); Ig0=zeros(4,1); IgFct=zeros(Nct,4);

dIdt=zeros(4,1);
I=zeros(4,1);
Stop=Nct;

Hill=(2*rand(HillCt,1))-1;

Iang=(Mwheel/8)*(Dw^2);
RPM=1e-9; % PWM=0;
ReachSpeed=0;
Iabcs=[0 0 0]';

%%%%%%%%%%%%%%%

MotorRndFct

RPM0=0; RPMg=0;
LastPosSSL=1;

omg=0; omgM=omg-(2*pi/3); omgP=omg+(2*pi/3);
Park=(2/3)*[cos(omg) cos(omgM) cos(omgP); sin(omg) sin(omgM) sin(omgP)

for ii=1:Nct
    fprintf([num2str(ii) '/' num2str(Nct) '\n']);
    t=t+dt;

    TopSpeed=(ceil(t/30))*10;
    if mod(ii,Nrng)==0
        MotorRndFct
    end

    T_drag=(Kdrag*2*Dw)*((RPM*pi/60)^2);

    Hill(1:(HillCt-1))=Hill(2:HillCt);
    Hill(HillCt)=(2*rand)-1;
    HillFct(ii)=mean(Hill);
    T_grav=(2*Mcar*grav/Dw)*(sin((AngRng*pi/180)*(mean(Hill))));
    CruiseRPM=(TopSpeed)*(5280/3.28)*(1/(60*pi*Dw));
```

```
if RPM<CruiseRPM
    if RPM<(CruiseRPM*(1-CruiseRng))
        Volt=Volt_max;
    else
        Volt=Volt_max*CruiseVolt;
    end
else
    if RPM<(CruiseRPM*(1+CruiseRng))
        Volt=Volt_max*CruiseVolt;
    else
        Volt=0;
    end
end
%Volt=Volt+(Volt_max/10);
U_m=Volt*eta_m;

f=P*RPM/(120*(1-slip));
if (f<fmin)
    f=fmin;
    slipFct(ii)=(fmin-(RPM/60))/fmin;
else
    slipFct(ii)=slip;
end
fFct(ii)=f;
omf=2*pi*f;
if dt>(1/PWM)
    ang=omf*t;
else
    dtpwm=mod((ii*dt),(1/PWM));
    ang=omf*(t-dtpwm);
end
angP=ang+(2*pi/3);
angM=ang-(2*pi/3);
Uabcs=(U_m)*[cos(ang); cos(angM); cos(angP)];
Uqds=Park*Uabcs;
UabcSfct(ii,:)=Uabcs;
I0=I;
Iabcs0=Iabcs;

[TorqueReal,I,Iabcs,Uqdo]=MotorFct(RPM,Uabcs,I,dt);
```

```
UqdFct(ii,:)=Uqdo;


% All Sensorless Material

if Volt==0
    FluxQD=FluxQD*0;
end
Irng=1+((RndRngI/100)*((2*rand)-1));
Ig(1:2)=I(1:2)*Irng;
for uu=1:2
    dFluxQD(uu)=Uqds(uu)-(R_s*I(uu));
    FluxQD(uu)=FluxQD(uu)+(dt*dFluxQD(uu));
    Ig(uu+2)=((FluxQD(uu))-(I(uu)*(M+L_ls)))/M;
    FluxQD(uu+2)=((Ig(uu+2))*(L_lr+M))+(M*(Ig(uu)));
end
FluxQDfct(ii,:)=FluxQD;
IgFct(ii,:)=Ig;


%%%%%%%%%%%

RPMg=SSL(Uabcs,Ig,Ig0);
if RPMg<=0
    RPMg=SSLfct(LastPosSSL);
else
    LastPosSSL=ii;
end
SSLfct(ii)=RPMg;

%U_qr=Uqdo(3)
%U_dr=Uqdo(4)

Ig0=Ig;
FluxQD0=FluxQD;

% End Sensorless Material


TorqueReal=abs(TorqueReal);
```

```
    AccAng=(TorqueReal+T_grav-T_drag)/Iang;

    RPM=RPM+(AccAng*dt);
    if RPM<0
        RPM=RPM0;
    end

    V=(RPM*pi*Dw/60)*(3600*3.28/5280);
    Vssl=(RPMg*pi*Dw/60)*(3600*3.28/5280);
    if Vssl>1e9
        Vssl=V0ssl;
    end

    X=X0+((V+V0)*dt/2);
    Xssl=X0ssl+((Vssl+V0ssl)*dt/2);
    X0=X; V0=V; X0ssl=X0; V0ssl=Vssl;
    if RPMg==0
        ErrorV(ii)=-1;
        ErrorX(ii)=-1;
    else
        ErrorV(ii)=abs((RPM-RPMg)/RPM);
        ErrorX(ii)=abs((X-Xssl)/X);
    end
    Xfct(ii)=X;
    XfctSSL(ii)=Xssl;
    Vfct(ii)=V;
    RPMfct(ii)=RPM;
    Afct(ii)=AccAng;
    VoltFct(ii)=Volt;
    TorqueRealFct(ii)=TorqueReal;
    Ifct(ii,:)=I;
    IabcSfct(ii,:)=Iabcs;
    angFct(ii)=sin(ang);

end
VfctSSL=(SSLfct*pi*Dw/60)*(3600*3.28/5280);
NanRangeSSL=find((SSLfct==SSLfct)==0);
runtime=toc;
save(DataFileName)
```

RawFct.m

```
function RawFct(PWM,RndRng,RndRngE,RndRngI,slip,fmin,DataFileName)

dt=5e-3; % Time Step (s)
TimeSim=5*60;
Nct=ceil(TimeSim/dt);
Nrng=round(Nct/5); %Nrng=floor(Nct/25);

%PWM=2000;
V00=0*(5280/(3.28*3600));
X00=0;

P=6;          % Number of Poles
eta_m=99/100;  % rotor shaft diameter
Volt_max=10000; % (Volts)
CruiseRng=0.05;
CruiseVolt=0.25;

R_r=15;         % Rotor Resistance (Ohm)
R_s=15;         % Stator Resistance (Ohm)
Ns=500;         % Number of loops (Stator)
Nr=500;         % Number of loops (Rotor)
RR=1e4;         % Henry^-1
k=0.086;        % Leakage

iter=10;

%%%%%%%%%%%%%%%% Car Properties

Mcar=1.5e3; % Mass of car = 1.5 metric tons
Dw=18*2.54/100; % Diameter of wheels = 18"
Mwheel=20; % Mass of the two wheels
Kdrag=2e0; % Drag coefficient of car

AngRng=30;
HillCt=0.1*round(Nct/TimeSim);
grav=9.81;
```

```
%%%%%%%%%%%%%%%%

L_ms=(Ns^2)/RR;         % (Henry) Stator Magnetizing Inductance
L_rs=Ns*Nr/RR;             % (Henry) Rotor / Stator Inductance
L_mr=(Nr^2)/RR;          % (Henry) Rotor Magnetizing Inductance

L_ls=L_ms*k;                 % (Henry) Stator Leakage Inductance
L_lr=L_mr*k;                  % (Henry) Rotor Leakage Inductance

M=(3/2)*L_ms;
L_SM=L_ls+M;
L_RM=L_lr+M;

%%%%%%%%%%%%%%%%

save RawData

end
```

MotorFct.m

```
function [Torque,I,Iabcs,Uout]=MotorFct(RPM,U_abcs,I0,dt)

load RawData

load MotorRndDat

% -------------------------------------------
% I(1) = i_qs; I(2) = i_ds; I(3) = i_qr; I(4) = i_dr

omr=2*pi*RPM/60;

omg=0;              % Set to 0 for relative to stationary plane
omgM=omg-(2*pi/3); omgP=omg+(2*pi/3);
M=(3/2)*L_ms;
L_SM=L_ls+M;
L_RM=L_lr+M;

Iqs=I0(1);
Ids=I0(2);
Iqr=I0(3);
Idr=I0(4);

A = [(-L_RM*R_s) 0 (M*R_r) 0; 0 (-L_RM*R_s) 0 (M*R_r); (M*R_s) 0 (-L_S[illegible]
C = [L_RM 0 -M 0; 0 L_RM 0 -M; -M 0 L_SM 0; 0 -M 0 L_SM];
A=A/((L_SM*L_RM)-(M^2)); C=C/((L_SM*L_RM)-(M^2));

Park=(2/3)*[cos(omg) cos(omgM) cos(omgP); sin(omg) sin(omgM) sin(omgP)[illegible]
U_qdos=Park*U_abcs;
u_qs=U_qdos(1); u_ds=U_qdos(2);
```

```
I=I0;
dIdt=zeros(4,1);
for ii=1:iter
    B = [-M*((M*Ids)+(L_RM*Idr)); M*((M*Iqs)+(L_RM*Iqr)); L_SM*((M*Ids)
    B=B/((L_SM*L_RM)-(M^2));

    u_qr=(R_r*Iqr)+((-omr)*((L_lr*Idr)+(M*Ids)+(M*Idr)))+(L_lr*dIdt(3))
    u_dr=(R_r*Idr)+(omr*(L_lr*Idr))+(omr*(M*Iqs))+(omr*M*Iqr)+(L_lr*dId
    U=[u_qs; u_ds; u_qr; u_dr];

    dIdt=(A*I)+(B*omr)+(C*U);
end

I(1)=I0(1)+(dt*dIdt(1));
I(2)=I0(2)+(dt*dIdt(2));
I(3)=I0(3)+(dt*dIdt(3));
I(4)=I0(4)+(dt*dIdt(4));

if max(abs(U_abcs))==0
    Torque=0;
    I=zeros(4,1);
    Iabcs=zeros(3,1);
else
    Torque=(3*P*M/4)*((Iqs*Idr)-(Ids*Iqr));
    Iqds=[I(1); I(2); 0];
    Iabcs=Iqds\Park; Iabcs=Iabcs';
end
Uout=U;

end
```

MotorRndFct.m

```
function MotorRndFct

load RawData

R_r=R_r*(1+((RndRng/100)*((2*rand)-1)));
R_s=R_s*(1+((RndRng/100)*((2*rand)-1)));
L_ms=L_ms*(1+((RndRng/100)*((2*rand)-1)));
L_ls=L_ls*(1+((RndRng/100)*((2*rand)-1)));
L_lr=L_lr*(1+((RndRng/100)*((2*rand)-1)));

save MotorRndDat R_r R_s L_ms L_ls L_lr

end
```

SSL.m

```
function [RPM]=SSL(UabcsX,Ix,I0)

load RawData

Erng=1+((RndRngE/100)*((2*rand)-1));
Irng=1+((RndRngI/100)*((2*rand)-1));
Uabcs=UabcsX*Erng; I=Ix*Irng;

omg=0;              % Set to 0 for relative to stationary plane
omgM=omg-(2*pi/3); omgP=omg+(2*pi/3);
M=(3/2)*L_ms;
L_SM=L_ls+M;
L_RM=L_lr+M;

Iqs=I0(1);
Ids=I0(2);
Iqr=I0(3);
Idr=I0(4);

A = [(-L_RM*R_s) 0 (M*R_r) 0; 0 (-L_RM*R_s) 0 (M*R_r); (M*R_s) 0 (-L_S[illegible]
C = [L_RM 0 -M 0; 0 L_RM 0 -M; -M 0 L_SM 0; 0 -M 0 L_SM];
A=A/((L_SM*L_RM)-(M^2)); C=C/((L_SM*L_RM)-(M^2));

Park=(2/3)*[cos(omg) cos(omgM) cos(omgP); sin(omg) sin(omgM) sin(omgP)[illegible]
U_qdos=Park*Uabcs;
u_qs=U_qdos(1); u_ds=U_qdos(2);

dIdt=(I-I0)/dt;

%%%%%%%%%%%%%%%%%%%%%%%%
```

```
A = [(-L_RM*R_s) 0 (M*R_r) 0; 0 (-L_RM*R_s) 0 (M*R_r); (M*R_s) 0 (-L_SM
B = [-M*((M*Ids)+(L_RM*Idr)); M*((M*Iqs)+(L_RM*Iqr)); L_SM*((M*Ids)+(L_
C = [L_RM 0 -M 0; 0 L_RM 0 -M; -M 0 L_SM 0; 0 -M 0 L_SM];
A=A/((L_SM*L_RM)-(M^2)); B=B/((L_SM*L_RM)-(M^2)); C=C/((L_SM*L_RM)-(M^2

u_qr=(R_r*Iqr)+(L_lr*dIdt(3))+(M*dIdt(1))+(M*dIdt(3));
u_qr2=((-1)*((L_lr*Idr)+(M*Ids)+(M*Idr)));

u_dr=(R_r*Idr)+(L_lr*dIdt(3))+(M*dIdt(1))+(M*dIdt(3));
u_dr2=(((L_lr*Idr)+((M*Iqs))+(M*Iqr)));

U1=[u_qs; u_ds; u_qr; u_dr]; U2=[0; 0; u_qr2; u_dr2];
omrG=(dIdt-(A*I0)-(C*U1)).*((B+(C*U2)).^-1);
omrG=omrG(2);

logic=omrG==omrG;
if logic==0
    omrG=0;
end


RPM=omrG/(2*pi/60);

end
```

ForceExt.m

```
function [Force_External] = ForceExt(t)

load RawData

A=(Aflex*sin(4*pi*t/TimeSim))+((Aflex/10)*((2*rand)-1));

if (abs(Aflex)>0)
    if (t>(TimeSim/4))
        if (t<(TimeSim/2))
            A=A+(-Adesign/25);
        end
    end
end

Force_External=Mplane0*A;

end
```
